# Supplementary material for: Comparison of Mineral, Metabolic, and Oxidative Profile of Saanen Goat during Lactation with Different Mediterranean Breed Clusters under the Same Environmental Conditions
Source: Animals (Basel). 2020 Mar 4;10(3):432. doi: 10.3390/ani10030432 (PMC7175111; doi:10.3390/ani10030432)
Supplement: Supplementary file 1 [file animals-10-00432-s001.pdf]

Article

Supplementary Materials: Comparison of Mineral, Metabolic, and Oxidative Profile of Saanen Goat during Lactation with Different Mediterranean Breed Clusters under the Same Environmental Conditions

Carmen L. Manuelian [1], Aristide Maggiolino [2,*], Massimo De Marchi [1], Salvatore Claps [3], Luigi Esposito [4], Domenico Rufrano [3], Elisabetta Casalino [2], Alessandra Tateo [2], Gianluca Neglia [4] and Pasquale De Palo [2]

[1] Department of Agronomy, Food, Natural resources, Animals and Environment, University of Padova, Viale dell'Università 16, 35020 Legnaro, Italy; carmenloreto.manuelianfuste@unipd.it (C.L.M.); massimo.demarchi@unipd.it (M.D.M.);

[2] Department of Veterinary Medicine, University of Bari Aldo Moro, Casamassima km 3, 70010 Valenzano , Italy; elisabetta.casalino@uniba.it (E.C.); alessandra.tateo@uniba.it (A.T.); pasquale.depalo@uniba.it (P.D.P);

[3] Council for Agricultural Research and Agricultural Economy Analysis - Research Centre for Animal Production and Aquaculture, S.S.7 Via Appia, 85051 Bella Muro, Italy; salvatore.claps@crea.gov.it (S.C.); drufrano@tiscali.it (D.R);

[4] Department of Veterinary Medicine and Animal Production (DMVPA), University of Naples Federico II, Naples, Via Federico Delpino 1, 80137 Napoli, Italy; luigespo@unina.it (L.E.) neglia@unina.it (G.N.);

* Correspondence: aristide.maggiolino@uniba.it; Tel.: +39-0805449915

Received: 17 February 2020; Accepted: 27 February 2020; Published: date

Table S1. Monthly pasture botanical composition during Winter (February-March), Spring (April, May, June), Summer (July, August), Autumn (September, October).

	Winter	Spring	Summer	Autumn
Medicago polimorfa	45.2 ± 8.6	18.9 ± 6.4	9.8 ± 2.3	12.1 ± 3.1
Lolium perenne	20.6 ± 5.2	17.1 ± 3.2	4.3 ± 0.7	14.3 ± 3.7
Bromus spp.	7.1 ± 2.2	4.4 ± 1.1	0	2.2 ± 0.5
Dactylis glomerata	4.4 ± 1.1	4.4 ± 1.0	2.2 ± 0.7	0
Phleum pratense	4.4 ± 0.8	0	0	2.2 ± 0.5
Agrostis stolonifera	3.3 ± 0.7	5.5 ± 1.3	1.1 ± 0.2	2.2 ± 0.5
Festuca arundinacea	2.3 ± 0.8	2.2 ± 0.5	0	8.8 ± 2.3
Aspp.erula odorata	2.2 ± 0.4	3.3 ± 0.8	2.2 ± 0.5	0
Geranium spp..	2.1 ± 0.5	6.5 ± 1.6	0	2.2 ± 0.5
Trifolium spp.	1.3 ± 0.3	3.3 ± 0.8	2.2 ± 0.5	18.1 ± 4.9
Cichorium intybus	1.2 ± 0.3	2.2 ± 0.5	7.4 ± 1.8	3.3 ± 0.8
Muscari neglectum	1.2 ± 0.3	1.1 ± 0.2	0	1.1 ± 0.2
Ranunculus bulbosum	1.2 ± 0.3	2.2 ± 0.5	1.1 ± 0.2	0
Rumex spp.	1.0 ± 0.2	1.1 ± 0.2	1.1 ± 0.2	4.2 ± 0.8
Veronica persica	0.8 ± 0.2	1.1 ± 0.2	0	0
Avena barbata	0	3.3 ± 0.8	1.1 ± 0.2	0
Hordeum vulgaris	0	2.7 ± 0.7	0	0
Borago officinalis	0	2.7 ± 0.7	0	0
Plantago spp.	1.7±0.4	2.7 ± 0.5	2.7 ± 0.7	4.3 ± 1.3
Poa pratensis	0	1.8 ± 0.4	0	0

Vicia sativa	0	1.8 ± 0.5	0	0
Foeniculum vulgare	0	1.8 ± 0.4	3.6 ± 0.9	3.4 ± 0.7
Galium verum	0	1.8 ± 0.3	1.8 ± 0.4	0
Mentha arvensis	0	1.8 ± 0.3	2.7 ± 0.7	0
Lotus corniculatus	0	0.9 ± 0.3	1.8 ± 0.4	0
Melilotus sulcatus	0	0.9 ± 0.1	1.8 ± 0.4	4.5 ± 1.0
Crepis spp..	0	0.9 ± 0.2	6.3 ± 1.1	0
Chrysantemum spp.	0	0.9 ± 0.2	1.8 ± 0.3	0
Daucus carota	0	0.9 ± 0.2	3.6 ± 1.2	3.6 ± 0.8
Malva sylvestris	0	0.9 ± 0.2	0.9 ± 0.1	0.9 ± 0.2
Picris spp.	0	0.9 ± 0.2	15.3 ± 3.7	0
Polygonum aviculare	0	0	9.9 ± 2.4	0
Cinorosus cristatus	0	0	2.7 ± 0.6	2.7 ± 0.6
Convonvolus arvensis	0	0	2.7 ± 0.6	0
Salvia viridis	0	0	2.7 ± 0.6	0
Agropyron oelungatum	0	0	1.8 ± 0.4	1.8 ± 0.3
Lotus corniculatus	0	0	1.8 ± 0.4	0
Centaurea spp.	0	0	1.8 ± 0.44	0
Thymus vulgaris	0	0	1.8 ± 0.4	2.7 ± 0.5
Sonchus spp..	0	0	0	5.4 ± 1.2

Data reported as arithmetic mean ± standard deviation (*n* = 9) in grams

Table S2. Monthly pasture chemical and nutritional composition during Winter (February-March), Spring (April, May, June), Summer (July, August), Autumn (September, October). arithmetic mean ± standard deviation; *n* = 9).

	**Winter**	**Spring**	**Summer**	**Autumn**
Dry Matter (g/kg as fed)	247.6 ± 45.5	264 ± 52.8	490.8 ± 103.0	246.4 ± 39.4
Organic Matter (g/kg DM)	896.9 ± 134.5	955.5 ± 124.2	926.8 ± 148.2	930.8 ± 158.2
Crude protein (g/kg DM)	173.9 ± 22.8	225.6 ± 33.8	114.3 ± 17.1	163.8 ± 26.2
Ether extract (g/kg DM)	27.8 ± 7.6	31.5 ± 8.5	20.1 ± 5.829.0	29.8 ± 8.9
NDF (g/kg DM)	419.6 ± 26.9	461.2 ± 46.1	389.3 ± 38.9	393.4 ± 78.6
ADF (g/kg DM)	356.2 ± 24.5	391.7 ± 39.1	329.4 ± 32.9	334.7 ± 66.9
ADL (g/kg DM)	6.1 ± 2.6	5.4 ± 2.1	15.2 ± 4.5	12.8 ± 5.1
Nutritive value (FUM/kg DM)	0.81 ± 0.1	0.89 ± 0.1	0.65 ± 0.1	0.82 ± 0.1

FUM = feed units for milk; NDF = neutral detergent fiber; ADF = acid detergent fiber; ADL = acid detergent lignin; DM = dry matter

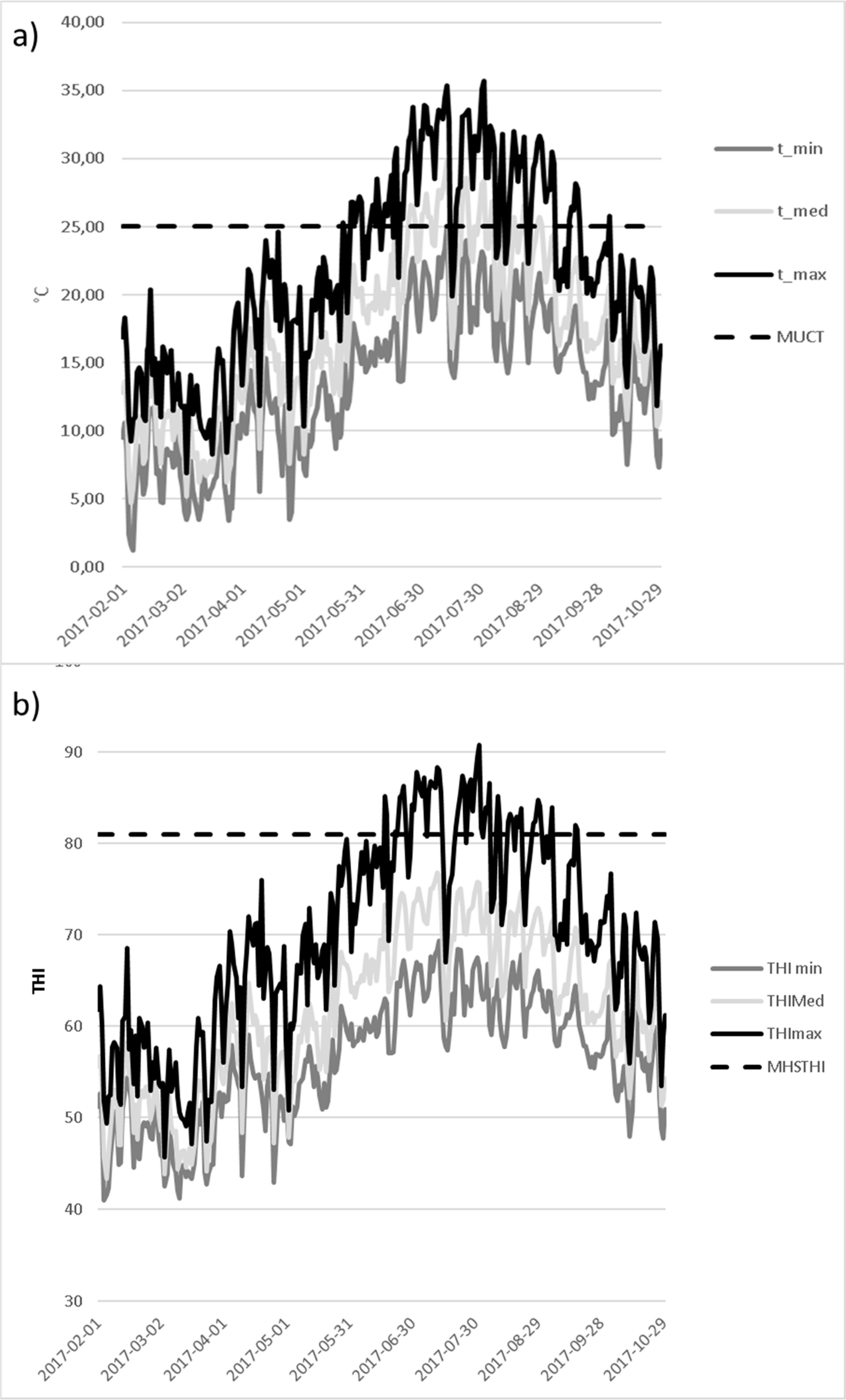

Figure S1. Daily maximum (t_max), minimum (t_min), and medium (t_med) temperatures (a) and temperature humidity index (THI) values (b) during the trial period, with the indication of minimum upper critical temperature (MUCT) according to Lu [14] and moderate heat stress THI (MHSTHI) according to Sano et al.[15] , respectively.
